# Race and gender biases in assessing pain intensity and medication needs among Chinese observers

**DOI:** 10.1097/PR9.0000000000001231

**Published:** 2024-12-26

**Authors:** Zhiyuan Liu, Tzu-Ying Chuang, Shan Wang

**Affiliations:** a Division of Natural and Applied Sciences, Duke Kunshan University, Kunshan, China; b Department of Social and Behavioral Sciences, Yale School of Public Health, New Haven, CT, USA; c Queen Square Institute of Neurology, University College London, London, United Kingdom; d Global Health Research Centre, Duke Kunshan University, Kunshan, China; e Department of Psychology & Centre for Pain Research, University of Bath, Bath, United Kingdom

**Keywords:** Pain assessment, Race, Gender, Stereotypical beliefs, Facial expressions

## Abstract

**Introduction:**

Pain-related decision-making can be influenced by the caregiver and sufferer's demographic factors, such as race and gender, which are commonly considered individually. However, such factors may influence pain assessment interdependently based on caregivers' stereotypical beliefs.

**Objectives:**

This study investigated how sufferers' race and gender affect Chinese observers' evaluations of pain intensity and medication needs and the associations with the observers' race and gender-related stereotypical beliefs.

**Methods:**

One hundred sixty-two Chinese participants completed a pain facial expression rating task assessing the level of pain intensity and the extent to which they believe the sufferer needs pain medication for East Asian, White, and Black sufferers, followed by a battery of questionnaires measuring their gender and race role expectation of pain.

**Results:**

Chinese observers were more stringent on East Asians' pain intensity and needs for medication than Whites and Blacks'. However, when Black and East Asian sufferers were perceived to have a similar level of pain, East Asians were more likely to receive medication than Blacks, which is partly due to the observers' stereotypical beliefs that Blacks would have a speedier recovery than East Asians.

**Conclusions:**

Our results provided evidence that in addition to the in-versus-out-group preferences, one's sociocultural-related stereotypical beliefs can account for racial disparities in pain-related decision-making.

## Abstract 2

Supplemental Digital Content is Available in the Text.

Native Chinese observers are more stringent when assessing East Asian sufferers' pain compared with Black and White sufferers'.

## 1. Introduction

Demographic factors affect individuals' pain experiences. Black people and women often exhibit greater sensitivity and lower tolerance to pain.^19,23^ On the other hand, sufferers' demographic factors, such as race and gender, also significantly influence how others perceive their pain.^2,4,14,42,46,48^ For example, racial minority groups (eg, Black and Hispanic patients in the United States)^1,12,24,26^ and female patients are found to be more vulnerable to the underestimation and suboptimal treatment of pain^12,15,27,36,50^ (but see Ref. 13). Studies so far have primarily focused on individual factors, eg, how either race or gender accounts for disparities in pain assessment and management. When considering sufferers' race, beliefs about the biological differences between Blacks and Whites, such as Blacks being physically tougher and experiencing less pain, led to assessment and treatment disparities.^16,43^ In clinical environments, health professionals who believed Black patients were less cooperative or compliant were also more likely to provide inadequate treatment recommendations to Black compared with White patients.^11,35^ When considering gender in pain-related behavior, women are typically viewed as more sensitive and reactive to noxious stimuli,^7,33^ more willing to express,^33,49^ and more likely to exaggerate,^21,31^ catastrophize,^22^ and dramatize pain than men.^25^ Regardless of whether true to fact or not, such gender stereotypical beliefs skew pain assessment, leading to a lower estimation of women's pain when both genders displayed similar pain expressions.^50^

Although often being considered in isolation, the influences of race and gender on pain-related decision-making are inseparable, particularly for facial expressions of pain, where race and gender-related cues are shown simultaneously. Studies found that the stereotypical associations mediate the perception of race and gender of faces.^17,40^ For example, the typical conceptual knowledge of Blacks as a race includes strength, dominance, and aggressiveness, aligning with the typical conceptual knowledge of men. Therefore, the perception of Black men's faces is facilitated by the shared conceptual knowledge between race and gender. By contrast, the typical conceptual knowledge of women is more sensitive, less tolerant, and tender. Thus, the perception of Black women's faces is impaired due to the conflicting stereotypical features between race and gender. Given that race and gender-related disparities in pain management are linked to stereotypical beliefs, it is thus important to consider how race and gender jointly influence pain appraisal. A recent study examined the perception of pain expressions presented by Black and White male and female faces but failed to replicate the sociocultural biases.^8^ One possible reason is that the facial stimuli were computer-generated race and gender prototypes, in which the sociodemographic features may be perceived as less realistic. Therefore, the primary research question of this study is to examine how sufferers' race and gender interdependently influence the assessment of pain intensity and medication needs using real-human facial expressions (Q1). We also examined the role of observers' sex. As suggested by Weisse et al.,^47,48^ gender and racial differences were evident only when the role of physicians' sex was taken into account. In this study, the term sex refers to observers' biological categorization of men and women assigned at birth, and *gender* refers to pain sufferers being perceived as men or women by observers. Detailed discussions on the distinctions and overlaps between the terms sex and gender are beyond the scope of this study (please refer to Refs. 19, 20 for reviews).

Previous work considering racial disparities and stereotypes has been primarily established in the U.S. context, focusing on comparing Black vs White sufferers with observers or caregivers being Whites. Less is known about other races or cultures, eg, East Asian (EA). Studies found that EAs' (Chinese) mental representation of pain expressions is different from Westerners' (British and Canadian).^5,37^ However, whether such differences are associated with sufferers' race and whether it would lead to disparities in pain assessment remains unknown. Among work examining empathy for pain in Chinese samples, racial-in vs racial-out group comparisons have been focused.^39,52^ However, given that both male and female observers tended to underestimate female sufferers' pain to a greater extent,^50^ and both White and Black caregivers believed Black patients feel less pain,^43^ the in-versus-out-group preferences may not be the leading cause of such biases, but the stereotypical beliefs. Thus, in this study, we investigated Chinese observers' appraisal of Black, White, and EA sufferers' pain and sought to explore to what extent they would hold and rely on race and gender stereotypes to perceive and evaluate others' pain and medication needs through facial expressions (Q2).

In terms of sufferers' race, we expected to observe a similar Black-White racial disparity, with Black sufferers receiving lower pain estimation and less pain medication than White sufferers. A recent study found that EAs (Chinese) would expect more intense facial expressions in the decoding of pain as compared with Westerners,^37^ which mirrors a tendency among EAs to only express pain overtly when it becomes unbearable.^37,45^ Whether such effect was moderated by sufferers' race is to be explored. Given that one's mental representation was formed by personal experiences and prior knowledge that is familiar to the individual, when sufferers' race is considered, EA observers' stringent threshold of pain intensity discrimination may be exaggerated for EA sufferers compared with other races. Therefore, we hypothesized that Chinese observers would adopt an even higher threshold and result in a lower pain appraisal for EAs than for other racial groups. When considering the joint influence of race and gender, a stereotypical association has been established between EA and female gender in both Western cultures and among those who are native to China,^3^ making EA women more prototypical of the racial group^38^ and suffer from intersectional discrimination on health-related issues.^9^ We, therefore, assumed that EA women would be more vulnerable to the stringent threshold on pain evaluation, and such potential biases are expected to be associated with the observers' race and gender-related stereotypical beliefs.

We also sought to disentangle whether race and gender-related biases in pain management are purely due to the biases in assessment or whether such biases can influence medication needs appraisal in addition to pain assessment (Q3). It was found that even with similar pain complaints in the medical records, Black patients were less likely to receive analgesics than White patients for extremity fractures.^41^ Thus, in this study, we assumed that race and gender-related biases would still exist in the appraisal of medication needs after controlling for pain intensity estimation, and such biases might be related to one's stereotypical beliefs.

## 2. Methods

### 2.1. Participants

One hundred eighty-three Mandarin-speaking Chinese adults were initially recruited at Duke Kunshan University, followed by a snowball sampling to the public. Participants were included if they had a normal or correct-to-normal vision and reported being pain-free and free from known psychological and neurological disorders. Data from 21 participants were excluded due to 19 failing attention checks and 2 having errors in data entry. The final sample included 162 participants (105 women) aged between 18 and 51 years (mean = 23.20).

### 2.2. Stimuli

A total of 48 face images presented by 24 models were selected as stimuli from the Delaware Pain Database (DPD),^29^ with 4 male and 4 female models of each race—Black, White, and EA and 2 face images from each model. Although the DPD provides a set of computer-generated facial expressions, studies observed significant differences in processing computer-animated and real-human expressions, where stronger neural activities and more accurate emotion recognition were found for real-human faces.^6,30,51^ We, therefore, chose to use images of real-human faces from the DPD. The selection process and selected stimuli are listed in Supplement 1, http://links.lww.com/PR9/A277. We computed the objective Facial Action Coding System (FACS) score for each facial stimulus by using the reliable pain-relevant facial movements: AU4 (brow lowering), AU6 (cheek raising), AU7 (lid tightening), AU9 (nose wrinkling), and AU45 (blinking). An AU is a fundamental component that describes specific, observable movements of facial muscles. Each AU represents a distinct muscular action. The FACS score was used as a control variable in the analyses. The Pain Specificity score from the DPD, which indicates how distinctively the stimulus was perceived as showing pain to other emotions, was also used as a control variable in the analysis of this study. More details of FACS and Pain Specificity can be found in Supplement 2, http://links.lww.com/PR9/A277.

### 2.3. Procedure

**Figure 1.**
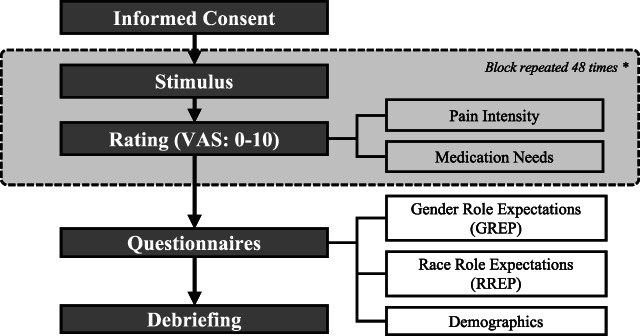
Flow chart of the experimental procedure.

This experiment was hosted online on *SoJump* (https://www.wjx.cn) and consisted of a rating task and a battery of questionnaires (Fig. 1). In the rating task, participants viewed 1 facial image at a time and assessed the level of the sufferer's pain intensity (0 = “no pain at all” to 10 = “extremely painful”) and the extent to which they believe the sufferer needs pain medication (0 = “no need at all” to 10 = “extremely needed”). Images were presented randomly across participants. To prevent rapid progression and ensure adequate viewing time, images remained visible for at least 3 seconds before participants could proceed to the next one. This task was followed by a battery of questionnaires measuring gender role expectation of pain and race/ethnicity role expectation of pain, after which participants completed a brief demographic questionnaire. The experiment took approximately 30 minutes to complete, and each participant received 20 CNY as compensation. The experiment protocol was reviewed and approved by the Institutional Review Board of Duke Kunshan University (#2021SW066).

### 2.4. Measures

#### 2.4.1. Gender role expectations of pain

A modified gender role expectations of pain (GREP) questionnaire of 6 items was used to examine participants' expectations of gender-related stereotypic attributions of pain sensitivity, pain endurance, and willingness to report pain for typical men and typical women.^34,46^ Items were rated on an 11-point visual analog scale (VAS) from −5 (not at all sensitive, no endurance at all, or not at all willing) to 5 (most sensitive imaginable, most endurance imaginable, or most willing imaginable), with 0 as a mid-point (typical men and women are perceived to be similar) for easier comparison. Similar to Zhang et al.,^50^ gender stereotype scores were derived by subtracting ratings for typical women from those for typical men, ranging from −10 (strongest bias in favor of thinking the typical women have higher pain sensitivity/endurance/willingness to report) to 10 (strongest bias in favor of thinking the typical men have higher pain sensitivity/endurance/willingness to report).

#### 2.4.2. Race/ethnicity role expectations of pain

Following Wandner et al. and Hoffman et al.,^16,46^ a modified Race/Ethnicity Role Expectations of Pain (RREP) questionnaire was used to measure race-related stereotypical attributions, including pain sensitivity, pain endurance, the willingness to report pain and the time length needed to recover from pain, across Black, White, and EA on an 11-point VAS from 0 (not at all sensitive, no endurance at all, not at all willing, or immediate recovery) to 10 (most sensitive, most endurance, most willing to report, or most lengthy recovery imaginable).

### 2.5. Data analysis

Data analyses were performed using R (The R functions and packages used in the analyses: Q1 linear mixed-effect models: lmer {lme4}, random effects significance test: ranova {lmerTest}; Q2 hierarchical comparisons of linear mixed-effect models: lmer {lme4}, anova {stats}; Q3 hierarchical regressions: lm {stats}, anova {stats}). Analyses began by examining the influences of race and gender on pain intensity estimation and appraisal of medication needs (Q1). Separate multilevel linear mixed models (in terms of the model assumptions, we examined the linearity [plotting residual against dependent variable], distribution [qq-plot] and homoscedasticity of residuals [plotting residuals again fitted model], as well as homogeneity of variance [Levene test]) were fitted, where sufferers' race and gender and observers' sex were the independent variables, and the centered FACS and Pain Specificity scores were covariates. The effect of FACS and Pain Specificity was also considered at the participant level as random slopes. Following Judd et al.,^18^ the random intercept effect was considered at the participant level and facial stimulus level. Wald *t*-tests were used to test the significance of each fixed effect in the model. The degree of freedom was calculated using Satterthwaite approximation.

To examine whether observers' stereotypical beliefs about race and gender could explain a proportion of the variance in sufferers' race and gender in pain estimation (Q2), where significant effects were found in the previous mixed models, hierarchical comparisons of linear mixed-effect models were conducted, with centered FACS and Pain Specificity (block 1), RREP or GREP measures (with each being entered individually to block 2) and sufferers' race or gender (block 3) being predictors, respectively. The random intercept effect was considered at the participant level and facial stimulus level. To account for possible racial biases, we computed the differences in RREP sensitivity, endurance, willingness to report, and time length needed to recover between every dyad of the races (eg, RREP sensitivity for Black—RREP sensitivity for EA) and fitted the model for each of the pairwise race comparisons (Black vs EA, Black vs White, and White vs EA). Analyses of GREP and RREP are reported in Supplements 3 and 4, http://links.lww.com/PR9/A277.

To investigate possible racial and gender biases in the appraisal of medication needs after controlling for pain intensity estimation and whether GREP and RREP could explain such biases (Q3), a series of hierarchical regressions were applied with the appraisal of medication needs regressing on pain intensity estimation (block 1) and sufferers' race and gender (block 2). Where a significant effect was found for sufferers' race or gender, a new set of hierarchical models was applied with medication appraisal regressed on pain intensity estimation (block 1), RREP or GREP measures (with each being entered individually to block 2), and sufferers' race or gender (block 3), respectively.

## 3. Results

### 3.1. Influences of race and gender on the decoding of pain (Q1)

#### 3.1.1. Pain intensity estimation

**Figure 2.**
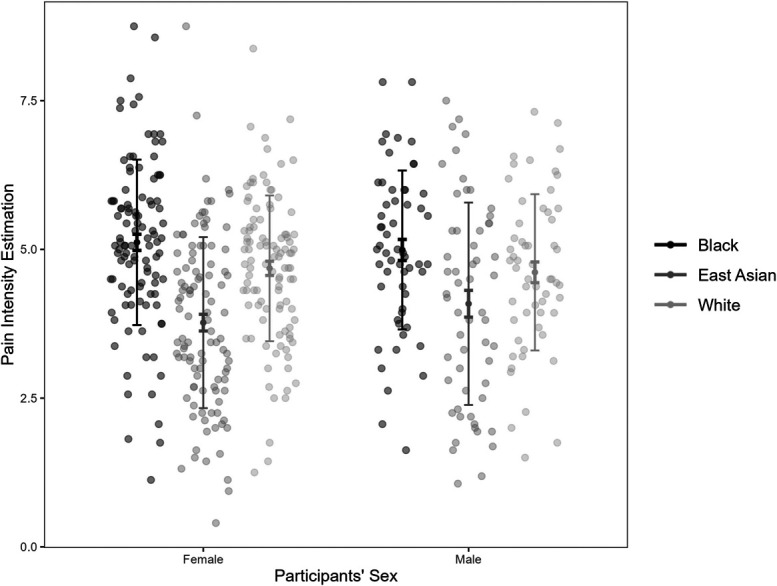
Female and male observers' pain intensity ratings for Black, East Asian, and White sufferers, with each dot showing a data point, the mid-point showing the mean, longer error bars showing the SD, and shorter error bars showing the SE.

Using a multilevel linear mixed model in R, we observed Pain Specificity had a significant random slope effect on participants' pain intensity estimation (*σ*^*2*^ = 0.21, χ^2^(3) = 218.10, *P* < 0.001), but FACS score did not (*σ*^*2*^ < 0.001, χ^2^(3) = 0.40, *P* = 0.94). The fixed effects of Pain Specificity and FACS score were not significant (both *P*s > 0.39). After controlling for FACS and Pain Specificity, a significant effect of the sufferer's race was found, where Black (*β* = 1.48, SE = 0.41, *t*(41.57) = 3.57, *P* < 0.001) and White faces' pain (*β* = 0.97, SE = 0.43, *t*(41.55) = 2.27, *P* = 0.029) was rated significantly higher than EAs'. The difference between Black and White sufferers' pain intensity was not significant (*β* = −0.51, SE = 0.40, *t*(41.64) = −1.30, *P* = 0.202). The interaction between sufferers' race and observers' sex was significant (Fig. 2). Moreover, male observers rated Blacks' pain more intense than EAs (*β* = 1.17, SE = 0.41, *t*(39.98) = 2.84, *P* = 0.007), but no significant differences between Whites' and other racial groups (both *P*s > 0.05). Female observers rated Black (*β* = 1.47, SE = 0.42, *t*(40.02) = 3.47, *P* = 0.001) and White faces' pain (*β* = 0.96, SE = 0.44, *t*(40.03) = 2.19, *P* = 0.035) more intense than EAs, but no significant difference between Black and White (*P* = 0.21). The main effect of the sufferer's gender, observer's sex, and other interactions were not significant (all *P*s > 0.09). The model assumptions were met (Levene test = 2.28, *P* = 0.13).

#### 3.1.2. Assessment of medication needs

**Figure 3.**
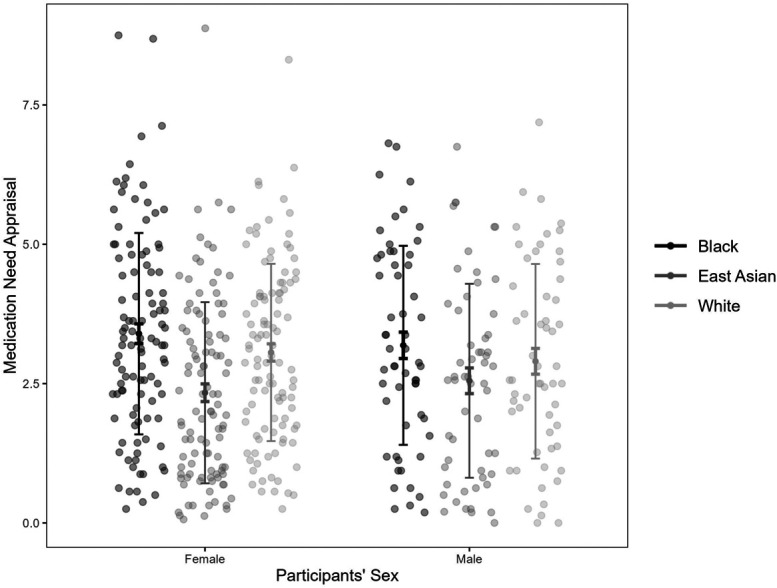
Female and male observers' medication needs appraisal for Black, East Asian, and White sufferers, with each dot showing a data point, the mid-point showing the mean, longer error bars showing the SD, and shorter error bars showing the SE.

Using a multilevel linear mixed model, we observed Pain Specificity had a significant random slope effect on participants' medication need appraisal (*σ*^*2*^ = 0.13, χ^2^(3) = 117.59, *P* < 0.001), but FACS score did not (*σ*^*2*^ = 0.003, χ^2^(3) = 6.75, *P* = 0.08). The fixed effects of Pain Specificity and FACS score were not significant (both *P*s > 0.30). After controlling for FACS and Pain Specificity, a significant effect of the sufferer's race was found, where Black (*β* = 1.19, SE = 0.33, *t*(42.26) = 3.58, *P* < 0.001) and White sufferers (*β* = 0.80, SE = 0.34, *t*(42.22) = 2.32, *P* = 0.025) were more likely to receive medication than EAs. The difference between Black and White sufferers' medication needs evaluation was not significant (*β* = −0.40, SE = 0.32, *t*(42.39) = −1.25, *P* = 0.220). The interaction between sufferers' race and observers' sex was significant (Fig. 3). Male observers believed Black sufferers needed more medication than EAs (*β* = 0.75, SE = 0.32, *t*(39.97) = 2.37, *P* = 0.023), but no significant differences between Whites' and other racial groups (both *P*s > 0.09). Female observers rated Black (*β* = 1.19, SE = 0.35, *t*(40.00) = 3.43, *P* = 0.001) and White faces' pain (*β* = 0.80, SE = 0.36, *t*(40.02) = 2.23, *P* = 0.032) more intense than EAs, but no significant difference between Black and White (*P* = 0.24). The main effect of the sufferer's gender, observers' sex, and other interactions were not significant (all *P*s > 0.13). The model assumptions were met (Levene test = 1.73, *P* = 0.19).

### 3.2. Stereotypical beliefs about race and gender related to the race and gender difference

Hierarchical comparisons of the linear mixed-effect models revealed that none of the RREP measures significantly accounted for Black-EA differences in pain intensity estimation (all −1.66 < *t*s < 0.76, *P*s > 0.099; χ^2^s < 2.71, *P*s > 0.100) or medication need appraisal (all −1.94 < *t*s < 0.26, *P*s > 0.054; χ^2^s < 3.66, *P*s > 0.056).

### 3.3. Racial and gender biases in the appraisal of medication needs after controlling for intensity estimation (Q3)

Hierarchical regressions revealed that after controlling for intensity estimation, the effect of race was marginally significant for the Black-EA comparison, where EA sufferers were more likely to receive pain medication than Blacks. A separate hierarchical regression revealed that this racial bias was significantly accounted for by the RREP—time length of recovery (Table 1). For Black-White and White-EA comparisons, the effect of race was no longer significant after controlling for pain intensity estimation (both *F* changes < 1.60, *R*^*2*^ changes < 0.001, *P*s > 0.20). Thus, no further analyses were conducted to examine the associated factors (Table 1).

**Table 1:** Results of hierarchical regressions on the racial bias between Black and Asian sufferers and the associated factor.

	*F* change	*R*^*2*^ change	*P—*Model comparison	Variables	*β*	*t*	*P*
Black vs Asian							
Race bias							
Model 1*	1106.25	0.631	<0.001	Intensity estimation	0.90	33.26	<0.001
Model 2	3.74	0.002	0.05	+ Race (Black vs Asian)	−0.18	−1.93	0.05
Race role expectations							
Model 1*	1106.25	0.631	<0.001	Intensity estimation	0.90	33.26	<0.001
Model 2	9.20	0.010	0.002	+ RREP-recovery time	−0.07	−3.04	0.002
Model 3	3.37	0.000	0.07	+ Race (Black vs Asian)	−0.17	−1.83	0.07
Black vs White							
Race bias							
Model 1*	867.30	0.576	<0.001	Intensity estimation	0.97	29.45	<0.001
Model 2	0.77	<0.001	0.38	+ Race (Black vs White)	−0.08	−0.88	0.38
White vs Asian							
Race bias							
Model 1*	1166.14	0.623	<0.001	Intensity estimation	0.90	31.97	<0.001
Model 2	1.82	<0.001	0.21	+ Race (White vs Asian)	−0.11	−1.26	0.21

* In each hierarchical regression analysis, model 1 is the base model. The *F*, *R*^2^, and *P*-values do not represent changes for model 1.

For gender-related biases, after controlling for intensity estimation, sufferers' gender difference was not significant (*F* change = 0.08, *R*^*2*^ change < 0.001, *P* = 0.77). Similarly, the Race × Gender effect was not significant for each of the race dyads (all *F* changes < 1.30, *R*^*2*^ changes < 0.003, *P*s > 0.27). No further analyses were conducted to examine the associated factors. The assumptions were met across all the models (Levene tests < 0.06, *P*s > 0.82).

## 4. Discussion

This study investigated: (1) how sufferers' gender and race influence Chinese layperson observers' estimation of others' pain intensity and medication needs through facial expressions, (2) whether observers' stereotypical beliefs about race and gender would account for potential race and gender biases, and (3) whether a gender or race-related disparity existed in the appraisal for medication need after controlling for pain intensity estimation.

### 4.1. Sufferers' race

We found that the sufferers' race significantly influenced the observers' estimation of pain. East Asian's pain was rated lower compared with sufferers of other races, even after controlling for the FACS and Pain Specificity of the facial stimuli. This supports our hypothesis that Chinese observers expect more intensely expressed pain according to their mental representation and tend to set a more stringent threshold for EA sufferers. Another possible reason that EA's pain is particularly underestimated may be due to the contradiction between observers' expectations and sufferers' expressions. Studies found that people's cultural-related beliefs of the “ideal” affect influence their social judgments of the target face and their emotional expressions.^32,44^ In our study, the Chinese observers’ RREP rating showed that EAs were expected to be less willing to express pain. However, according to the analysis of FACS scores, the EA facial stimuli used in this study showed as much pain as Black and White faces. Such contrast might have led Chinese observers to perceive the EA faces as less ideal or even exaggerating the pain, resulting in an even lower pain intensity rating.

We found that Black sufferers' pain was rated as intense as White sufferers' pain. This is in contrast to previous literature where a greater underestimation of Black sufferers' pain was often found in comparison with White sufferers' by White^16,24,28^ and sometimes Black observers.^14,16,28^ Given that the observers of this study were all native Chinese living in China, the contrast to previous findings implies that the disparity towards Black sufferers' pain may be rooted in the shared social culture environment where the studies were conducted. Future studies comparing Chinese or EA observers who are native to EA culture vs those grown up in Western culture may provide further evidence showing whether such biases are more sociocultural or racial.

In terms of the medication needs appraisal, race seemed to play a similar role as in the pain intensity estimation, where EA sufferers were perceived to have lower medication needs compared with Black and White sufferers. Indeed, the medication appraisal was significantly predicted by intensity rating (over 60% of the variance on average), showing that observers rely on their pain intensity rating to evaluate sufferers' medication needs. However, after controlling for the intensity rating, a racial bias was found between Black and EA sufferers—when Black and EA sufferers were perceived to experience a similar level of pain, EA sufferers were more likely to receive medication than Blacks. Such bias is partially due to the observers' race/ethnicity role expectations of pain recovering ability—EAs were believed to need more time to recover than Blacks. This is in line with a previous study indicating that false biological beliefs about differences between Blacks and Whites can lead to disparities in pain management,^16^ suggesting that racial bias in treatment recommendations could be attributed to observers' beliefs about race-related biological differences.

### 4.2. Sufferers' gender by race

Sufferers' gender did not affect the pain intensity rating and medication need appraisal. This is in contrast to previous findings that women's pain was more likely to be underestimated and undertreated than men's pain.^12,15,27,36,50^ Such gender-related disparities in pain management were considered due to the stereotypical beliefs about the differences between male and female sufferers. In this study, observers believed that typical women are more sensitive to pain and more willing to report pain than typical men, which is similar to the literature.^8,33,49^ However, such stereotypical beliefs did not lead to disparities in pain-related decision-making. One possible reason is that the facial stimuli used in this study were not perceived as “typical” men or women by the observers, so the observers' stereotypical beliefs of gender-related features may be less predictive of their pain assessment. To examine this, future studies shall consider measuring observers' perceptions of the sufferers' gender role typicality. Moreover, we did not find an intersectional effect of sufferers' race and gender in pain decoding, which did not support our hypothesis. One possible reason discussed above is a lack of gender effect. Another possible reason is a lack of facilitative or inhibitive effect between the stereotypical beliefs of race and gender. However, in this study, we considered the pain-related race and gender role expectations independently. A more comprehensive measure of GREP for different races or an RREP for women and men will help answer the question.

### 4.3. Observers' sex

Observers' sex was found to moderate the effect of sufferers' race. Female observers perceived both Black and White sufferers' pain to be more intense and needed more medication than EAs, whereas, for male observers, such difference was only found between Black and EA sufferers. We are unsure what caused such a difference. A possible factor to consider is the male and female observers' exposure to different races' pain and their expressions. In Western culture, exposure indicated by the racial diversity of the sampling region affects how Black and White faces are perceived,^10^ where higher exposure may reduce racial disparities. Given that racial diversity is limited in local Chinese communities, a self-report of exposure may be used in future studies.

In sum, this study found that Chinese observers are more stringent when assessing EA sufferers' pain compared with White and Black sufferers. Although Black sufferers' pain is rated as more severe, once the pain intensity level is controlled, ie, when Black and EA sufferers are perceived to have a similar level of pain, Black sufferers are less likely to receive medication than EAs. Such bias is partly due to the observers' stereotypical beliefs that Blacks tend to have a speedier recovery than EAs.

## Disclosures

The authors have no known conflict of interest to disclose.

## Supplementary Material

SUPPLEMENTARY MATERIAL

## Supplemental digital content

Supplemental digital content associated with this article can be found online at http://links.lww.com/PR9/A277.
