# Supplementary material for: Race and gender biases in assessing pain intensity and medication needs among Chinese observers
Source: Pain Rep. 2024 Dec 26;10(1):e1231. doi: 10.1097/PR9.0000000000001231 (PMC11677614; doi:10.1097/PR9.0000000000001231)
Supplement: SUPPLEMENTARY MATERIAL [file painreports-10-e1231-s001.pdf]

Supplemental Digital Content

1. Facial stimuli

1.1. The facial images included in the current stimuli set.

Target	Sufferer Race	Sufferer Gender	Model	Pain Specificity	FACS Score
DPD_1_AF16_p3c	Asian	Female	AF16	1.50	4
DPD_1_AF16_p5c	Asian	Female	AF16	1.31	5
DPD_1_AF17_p2c	Asian	Female	AF17	0.93	4
DPD_1_AF17_p6c	Asian	Female	AF17	0.18	4
DPD_1_AF23_p3c	Asian	Female	AF23	-0.78	4
DPD_1_AF23_p5c	Asian	Female	AF23	0.53	4
DPD_1_AF26_p2c	Asian	Female	AF26	0.46	4
DPD_1_AF26_p3c	Asian	Female	AF26	0.41	5
DPD_1_AM12_p2c	Asian	Male	AM12	1.60	3
DPD_1_AM12_p4c	Asian	Male	AM12	0.87	5
DPD_1_AM20_p4c	Asian	Male	AM20	1.79	4
DPD_1_AM20_p5c	Asian	Male	AM20	-1.04	3
DPD_1_AM5_p3c	Asian	Male	AM5	1.32	4
DPD_1_AM5_p5c	Asian	Male	AM5	1.93	5
DPD_1_AM7_p3b	Asian	Male	AM7	1.07	5
DPD_1_AM7_p5c	Asian	Male	AM7	2.17	3
DPD_1_BF16_p2c	Black	Female	BF16	0.60	4
DPD_1_BF16_p4c	Black	Female	BF16	1.52	4
DPD_1_BF17_p5c	Black	Female	BF17	0.62	4
DPD_1_BF17_p6c	Black	Female	BF17	1.55	4
DPD_1_BF21_p5c	Black	Female	BF21	1.51	5
DPD_1_BF21_p6c	Black	Female	BF21	1.95	5
DPD_1_BF29_p2c	Black	Female	BF29	1.96	4
DPD_1_BF29_p4b	Black	Female	BF29	0.67	4
DPD_1_BM14_p2c	Black	Male	BM14	1.07	5
DPD_1_BM14_p5c	Black	Male	BM14	1.76	5
DPD_1_BM23_p2c	Black	Male	BM23	1.28	3
DPD_1_BM23_p4c	Black	Male	BM23	2.57	4
DPD_1_BM2_p1	Black	Male	BM2	1.44	4
DPD_1_BM2_p2	Black	Male	BM2	2.05	3
DPD_1_BM4_p1	Black	Male	BM4	2.32	5
DPD_1_BM4_p3	Black	Male	BM4	2.08	4
DPD_1_WF16_p2c	White	Female	WF16	1.90	5
DPD_1_WF16_p5c	White	Female	WF16	0.50	4
DPD_1_WF27_p5c	White	Female	WF27	0.83	3
DPD_1_WF27_p6c	White	Female	WF27	1.66	4

DPD_1_WF2_p2c	White	Female	WF2	2.00	5
DPD_1_WF2_p5c	White	Female	WF2	2.02	4
DPD_1_WF5_p5c	White	Female	WF5	1.95	5
DPD_1_WF5_p6c	White	Female	WF5	1.27	5
DPD_1_WM14_p1	White	Male	WM14	3.07	5
DPD_1_WM14_p4	White	Male	WM14	1.62	5
DPD_1_WM43_p2c	White	Male	WM43	2.34	4
DPD_1_WM43_p5c	White	Male	WM43	2.08	4
DPD_1_WM5_p1	White	Male	WM5	2.79	5
DPD_1_WM5_p2	White	Male	WM5	1.23	5
DPD_1_WM6_p3	White	Male	WM6	1.62	4
DPD_1_WM6_p4	White	Male	WM6	2.72	5

1.2. The selection process for DPD stimuli

We aimed to select face stimuli from the DPD presented by Black, White and East Asian models.

We first considered the availability of the FACS score as a potential control variable. All the DPD stimuli had an FACS score automatically rated by software. However, only a proportion of the stimuli were also manually rated by researchers to examine rating reliability. We first selected DPD stimuli from the pool with the manual FACS rating available. The criteria used for selection are as below. First, we selected models whose self-report race was Black, White or East Asian. All the mixed-race models were excluded. Second, among these models, we selected the models whose self-report race matched with DPD norming observers' recognition of the model's race. For example, a self-report East Asian model must be recognized as an East Asian. We then checked each model to ensure each selected model had a Race Prototypicality score above the median of all models from that race category. Thirty faces were selected for the stage, including 8 East Asians (4 Females and 4 Males), 10 Blacks (4 Females and 6 Males), and 12 Whites (6 Females and 6 Males). Due to a smaller number of East Asian models in the DPD, to have the same number of models from each race and gender, we matched the number of Black and White models to East Asians and selected 8 Blacks (4 Females and 4 Males) and 8 Whites (4 Females and 4 Males) ranked higher in the Race Prototypicality score. We aimed to use two pain images from each model to have a reasonable number of stimuli per category (i.e., 8 images). However, among the 24 models, 5 models only had one pain image. We therefore excluded the 5 models: one East Asian (Female AF13), one Black (Female BF4), and two Whites (2 Females WF31, WF13). Among the selected models, we examined each face image and ensured their Pain Specificity scores were above the median of each category.

We then started another search of the DPD stimuli without manual FACS ratings to select another 5 models. The same selection criteria were applied by examining (1) whether the self-report race matched the observer-recognized race, (2) whether it was a single race, (3) the Race Prototypicality score above the median, (4) each model had two images, and (5) both images had a Pain Specificity score above the median of the race/gender category.

As a result, we selected 48 images from 24 models (three races, two genders, four models each), with two images from each model, as the facial stimuli in the current study.

Supplemental Digital Content

2. FACS and pain specificity score

2.1. FACS

In the DPD norming data, the AUs were coded as either active (score 1) or inactive (score 0). Thus, the computed FACS scores for the face stimuli range from zero to five. The FACS scores were entered into a 3 (sufferers' race) × 2 (sufferers' gender) ANOVA, and no significant differences were found across the sufferers' races and genders (all *F*s < 1.39, *p*s > .26).

2.2. Pain Specificity

The Pain Specificity scores were entered into a 3 (race) × 2 (gender) ANOVA. The main effects of sufferer's race ($F(2, 42) = 7.96$, $p = .001$, $\eta^2_p = .28$) and gender ($F(1, 42) = 9.26$, $p = .004$, $\eta^2_p = .18$) were significant. White sufferers' pain was more specific than Black and EA's (both $p_{corrected}$ *s* < .03), and males' pain was more specific than females'.

3. Gender role expectation of pain

We examined the internal consistency for each item of the gender role expectations of pain . One-sample t-tests (μ = 0) were used to examine gender stereotypes. A gender stereotype score significantly lower than zero indicated that women are believed to have higher pain sensitivity/endurance/willingness to report than men, and a score above zero indicated higher levels in men than women.

Internal consistency for each gender comparison was high (Cronbach's alpha: sensitivity = 0.925, endurance = 0.935, willingness to report = 0.919). One-sample *t*-tests (μ = 0) revealed significant gender stereotypes that women were believed to be more sensitive (mean = -1.99, sd = 4.34, $t(161) = -5.85$, $p < .001$) and more willing to report pain (mean = -3.84, sd = 4.42, $t(161) = -11.06$, $p < .001$) but not more endurable for pain (mean = -0.24, sd = 4.86, $t(161) = -0.63$, $p = .53$) than men.

4. Race/Ethnicity role expectations of pian

The data of race role expectations of pain was entered into four separate 3 (sufferers' race) × 2 (observers' sex) mixed ANOVAs. A significant main effect of sufferers' race was found for pain sensitivity ($F(1.90, 304.11) = 9.83$, $p < .001$), where Whites (mean = 5.70, sd = 1.61, $p < .001$) and East Asians (mean = 5.47, sd = 1.43, $p = .03$) were believed to be more sensitive to pain than Blacks (mean = 4.97, sd = 1.78). No significant difference was found between Whites and East Asians ($p = .25$). For pain endurance ($F(1.91, 305.96) = 45.66$, $p < .001$), White people (mean = 4.47, sd = 1.50) were believed to endure less pain than Blacks (mean = 5.71, sd = 1.86) and East Asians (mean = 5.96, sd = 1.67, both *p*s < .001). No significant difference was found between Blacks and East Asians ($p = .47$). For the willingness to report pain ($F(1.85, 295.55) = 54.12$, $p < .001$), where Whites (mean = 6.30, sd = 1.81) are believed to be more willing to report pain than Blacks (mean = 5.10, sd = 1.80) than East Asians (mean = 4.55, sd = 1.64, all *p*s < .002). A significant interaction of race × participants' sex was found for recovery time ($F(2, 320) = 4.60$, $p = .011$), where female participants believed East Asians (mean = 5.06, sd = 1.53) needed more time to recover than Blacks (mean = 4.38, sd = 1.72, $F(2, 208) = 8.00$, $p < .001$).

In RREP, each feature is unique, with one rating for each race, which does not allow us to analyze reliability as we did for GREP.
